# Serum Magnesium Levels in Preterm Infants Are Higher Than Adult Levels: A Systematic Literature Review and Meta-Analysis

**DOI:** 10.3390/nu9101125

**Published:** 2017-10-16

**Authors:** Jacques Rigo, Catherine Pieltain, Viola Christmann, Francesco Bonsante, Sissel J. Moltu, Silvia Iacobelli, Stéphane Marret

**Affiliations:** 1 Department of Neonatology, Université de Liège, CHR Citadelle, 4000 Liège, Belgium; catherine.pieltain@chu.ulg.ac.be; 2 Radboudumc Amalia Children’s Hospital, Radboud University Medical Center, 6500 HB Nijmegen, The Netherlands; Viola.Christmann@radboudumc.nl; 3 Centre d’Etudes Périnatales de l’Océan Indien (EA 7388), CHU La Réunion—Site Sud Saint Pierre, BP 350 97448 Saint Pierre CEDEX, France; francesco.bonsante@chu-reunion.fr (F.B.); silvia.iacobelli@chu-reunion.fr (S.I.); 4 Réanimation Néonatale et Pédiatrique, Néonatologie, CHU La Réunion—Site Sud Saint Pierre, BP 350 97448 Saint Pierre CEDEX, France; 5 Department of Neonatal Intensive Care, Oslo University Hospital, 0318 Oslo, Norway; smoltu@online.no; 6 Department of Neonatal Pediatrics and Intensive Care and Neuropediatrics, Rouen University Hospital, and INSERM, Laboratoire NeoVasc ERI28, Normandy University, 76000 Rouen, France; stephane.marret@chu-rouen.fr

**Keywords:** systematic literature review, meta-analysis, magnesium, neonates, cord blood, nutrition, supplementation

## Abstract

Magnesium (Mg) is an essential mineral in the body, impacting the synthesis of biomacromolecules, bone matrix development, energy production, as well as heart, nerve, and muscle function. Although the importance of Mg is evident, reference values for serum Mg (sMg) in pediatric patients (more specifically, in neonates) are not well established. This systematic literature review and meta-analysis (using 47 eligible studies) aims to quantify normal and tolerable ranges of sMg concentrations during the neonatal period and to highlight the factors influencing Mg levels and the importance of regulating sMg levels during pregnancy and birth. In newborns without Mg supplementation during pregnancy, magnesium levels at birth (0.76 (95% CI: 0.52, 0.99) mmol/L) were similar to that of mothers during pregnancy (0.74 (95% CI: 0.43, 1.04) mmol/L), but increased during the first week of life (0.91 (95% CI: 0.55, 1.26) mmol/L) before returning to adult levels. This pattern was also seen in newborns with Mg supplementation during pregnancy, where the average was 1.29 (95% CI: 0.50, 2.08) mmol/L at birth and 1.44 (95% CI: 0.61, 2.27) mmol/L during the first week of life. Factors influencing these levels include prenatal Mg supplementation, gestational age, birth weight, renal maturity/function, and postnatal Mg intake. Elevated Mg levels (>2.5 mmol/L) have been associated with an increased risk of mortality, admission into intensive care, hypotonia, hypotension, and respiratory depression but sMg concentrations up to 2.0 mmol/L appear to be well tolerated in neonates, requiring adequate survey and minimal intervention.

## 1. Introduction

Magnesium, the fourth most abundant cation in the body [1], plays a crucial role in many physiologic functions. It contributes to bone matrix development and is required for the synthesis of biomacromolecules, including DNA, RNA, and proteins [2,3,4]. It is needed for energy production and glycolysis [2,4,5,6]. Magnesium is also utilized for electrostatic stabilization in cell systems and participates in the regulation of active transport of calcium and potassium ions across cell membranes [7,8,9,10], thereby regulating muscle contraction, nerve impulse conduction, vascular tone, and normal heart function. Magnesium homeostasis is largely controlled by the kidney, with reabsorption occurring predominantly in the thick ascending limb of the loop of Henle (70–80%) and, to a lesser extent (10–15%), in the distal convoluted tubule [11]. It is regulated by many hormonal and nonhormonal factors, and reabsorption is closely linked to that of calcium [11].

Evaluation of serum magnesium (sMg) is the most common method of assessing magnesium concentration in clinical practice [12], despite the fact that it may not accurately reflect total body magnesium content—only approximately 1% of total body magnesium is in serum and interstitial body fluid, with the remainder in bone, muscle, and soft tissue [1]. Reference values for sMg in adults are well defined (0.75 mmol/L; 95% CI: 0.45, 1.05) [13,14]. By contrast, in pediatric patients—and more specifically in newborn and preterm infants (<37 weeks gestation [15])—reference values are either not available or are very limited and still controversial; most laboratories do not provide specific reference intervals for this population. 

Until recently, sMg concentration was scarcely included in the biological survey of newborn infants unless hypomagnesemia was suspected in association with persistent and refractory hypocalcemia [11]. Serum magnesium assessment has become more frequently requested due to the more regular early use of magnesium in balanced parenteral nutrition providing electrolytes and minerals for very-low-birth-weight (VLBW) infants; and the use of antenatal magnesium administration for neuroprotection in very preterm infants (<32 weeks gestation [15]) and/or for the prevention of eclampsia in preeclamptic mothers. Thus, there is a need for normative data and the determination of an upper tolerable level of sMg concentration in preterm and term infants. In 2012, the Canadian Laboratory Initiative in Pediatric Reference Intervals (CALIPER) project proposed new reference ranges for neonates that were developed using the CALIPER database, which included data from healthy infants and children in the community (1072 male and 1116 female; newborn to 18 years) [16]. CALIPER data suggested that sMg levels in healthy term infants during the first two weeks after delivery were higher than adult reference values and decreased progressively during the remainder of the first year of life to adult levels. Although analytical methods and reagents have changed since the time of this study, the provided ranges are useful for understanding changes in magnesium levels that occur during this time period.

Magnesium requirements in preterm infants remain poorly defined. Fetal accretion of magnesium occurs throughout pregnancy, with accretion rates reportedly ranging from 3 to 5 mg/kg/day (0.12–0.20 mmol/kg/day) during the third trimester [17]. Data derived from the chemical analysis of fetuses from 24 to 40 weeks of gestation demonstrated gradually increasing rates of magnesium accretion [18]. Preterm infants exhibit particularly high growth velocity, and they miss at least some of the period of high in utero accretion of magnesium; therefore, it is likely that magnesium requirements of preterm infants are different than those of infants delivered at full term.

The primary objectives of the current review are to examine magnesium levels in preterm infants during the neonatal period (0–28 days); explore major potential factors influencing magnesium concentration (i.e., gestational age, magnesium supplementation during pregnancy, postnatal magnesium intakes, and renal function) in an effort to better understand magnesium replacement requirements; and discuss reference sMg concentrations in preterm infants. As a secondary objective, this review evaluated the potential side effects of increased sMg in order to discern the upper tolerable levels in preterm infants during the first weeks of life. 

## 2. Materials and Methods

To find relevant published literature on magnesium levels, a search was conducted on PubMed that included terms for the type of patient (e.g., human, newborn, and infant), stage of care (e.g., prenatal, antenatal, and neonatal), and type of treatment/measures (e.g., magnesium, magnesium sulfate (MgSO_4_), nutrition, nutritional status, and nutritional sciences) and included articles published up to June 2014. A total of 322 articles were found. Article titles and abstracts were reviewed (3 contributors) for potential relevancy, and 143 were excluded; papers were excluded at this stage if they were review articles, if the study was in non-healthy infants, and if mineral concentrations were not a part of the focus. The full texts of the remaining 179 studies were reviewed and 51 were eligible as they reported magnesium levels in preterm and/or term infants before, at, and/or after birth. Studies were excluded if they reported mean magnesium levels that were clearly outliers among the collected data based on clinical expert opinion (JR); this included 4 studies [19,20,21,22]. 

The remaining 47 studies were used in meta-analyses to derive an estimated global mean and 95% confidence interval (CI) of the population concentration range using only studies reporting both mean and standard deviation data. Populations of interest included: healthy pregnant women without magnesium supplementation during pregnancy and healthy newborns with and without magnesium supplementation during pregnancy. In each meta-analysis, studies were weighted based on the number of sMg samples collected. In studies reporting both venous and arterial blood levels, the venous levels were used for the meta-analysis. Heterogeneity was quantified using the *I*^2^ statistic [23]. Magnesium data reported in non-SI units were converted to millimoles by multiplying by the appropriate conversion factor.

Additional information from other studies found from the reference lists of reviewed papers and articles not reporting exact magnesium levels was included to bolster the evidence. To increase the data available for preterm infants during the first week of life, the primary authors of four recent clinical studies in preterm infants (Drs. F. Bonsante, S. Iacobelli, S.J. Moltu, V. Christmann, and S. Marret) provided additional information on the sMg concentrations and/or intakes, as well as recorded during their studies [24,25,26,27,28,29]. This complementary information was included in the present manuscript.

Meta-analyses were conducted with OpenMeta[Analyst] (Brown University, Providence, RI, USA), a program funded by AHRQ (grant number: R01HS018574; available at http://www.cebm.brown.edu/openmeta/index.html), using a continuous random-effects model at the 95% confidence level.

## 3. Results

### 3.1. Maternal Magnesium Concentrations

In normal pregnancy, maternal sMg decreases. However, the exact time course of these changes has yet to be established [30]. Published data from pregnant women are presented in Table S1. In those studies, mean sMg concentration in mothers ranged from 0.59 mmol/L to 0.95 mmol/L during gestation [31,32,33,34,35,36,37,38,39,40,41,42,43,44,45], from 0.54 mmol/L to 0.86 mmol/L before or during labor [31,35,40,41,43,46,47,48,49], and from 0.54 mmol/L to 0.90 mmol/L at delivery [24,35,36,42,48,50,51,52,53,54,55,56,57,58,59,60,61,62,63].

**Figure 1:**
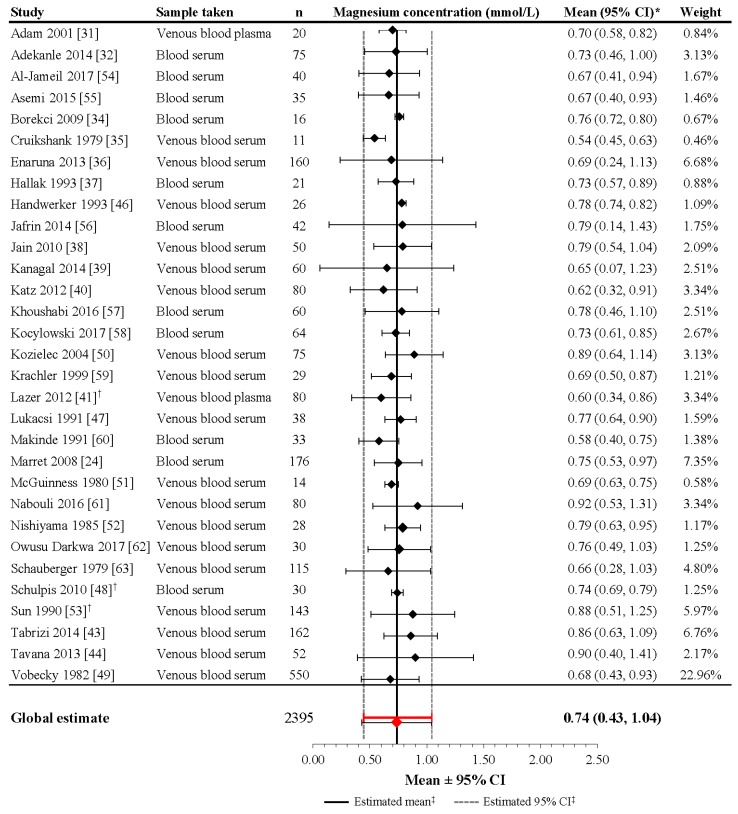
Magnesium concentrations in healthy pregnant women without magnesium supplementation during pregnancy. * If 95% CIs were not reported in the study, then the 95% CIs were calculated as follows: mean ± (1.96 × SD). ^†^ Patient groups within these studies were pooled, as the literature search did not confirm an association between magnesium levels and gestational age, birth weight, or mode of delivery. ^‡^ Reference line and 95% CI derived using only studies reporting both mean and SD data. Overall statistic for heterogeneity: *I*^2^ = 97.8% (*p* < 0.001). CI: confidence interval, SD: standard deviation.

Although the heterogeneity of the included studies was high (*I*^2^ = 99.6%; *p* < 0.001), the meta-analysis performed on 31 studies including 2395 mothers revealed a mean estimate of 0.74 mmol/L (95% CI: 0.43, 1.04) in pregnant women around delivery (Figure 1). For the studies not included in the meta-analysis [33,42,45], the mean (or median) reported magnesium level falls within the derived 95% CI, as well as the reported ranges.

Ranges of magnesium concentrations reported in healthy pregnant women without magnesium supplementation differ to the normal reference interval reflecting an adequate magnesium status for healthy adults (0.75–0.95 mmol/L) [64]. Changes in magnesium status in pregnant women may translate into effects on glucose metabolism, blood pressure, and the contractile response of uterine muscle. Thus, earlier reductions in maternal magnesium have been associated with an increased incidence of gestational diabetes [65,66,67] and may contribute to both preterm delivery and preeclampsia [36,68,69]. Few well-designed trials have examined the impact of dietary magnesium supplementation in pregnant women; thus, the benefits and risks of such remain controversial [70].

By contrast, MgSO_4_ supplementation in pregnant women experiencing preterm labor or preeclampsia has been more thoroughly studied in randomized trials. In these studies, the target sMg in the mother before delivery was between 2.0 and 3.5 mmol/L [71] and meta-analyses of these data suggest that these levels have a neuroprotective effect [72,73]. However, very few studies report the sMg concentrations in the mothers receiving MgSO_4_ supplementation during pregnancy (e.g., Marret et al. [24]).

### 3.2. Magnesium Levels at Birth in Neonates without Magnesium Supplementation during Pregnancy

**Figure 2:**
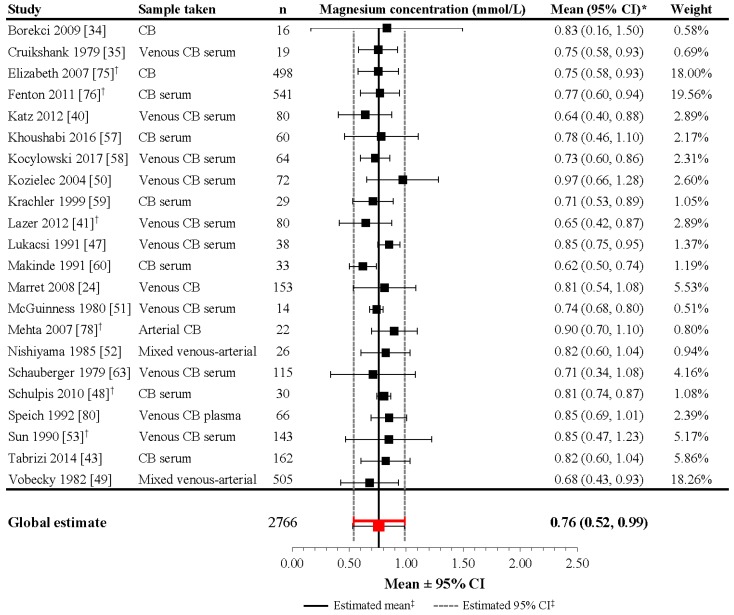
Magnesium concentrations in umbilical cord blood of healthy newborns without magnesium supplementation during pregnancy. * If 95% CIs were not reported in the study, then the 95% CIs were calculated as follows: mean ± (1.96 × SD). ^†^ Patient groups within these studies were pooled, as the literature search did not confirm an association between magnesium levels and gestational age, birth weight, or mode of delivery. ^‡^ Reference line and 95% CI derived using only studies reporting both mean and SD data. Overall statistic for heterogeneity: *I*^2^ = 98.3% (*p* < 0.001). CB: [umbilical] cord blood; CI: confidence interval; n: number of infants; SD: standard deviation.

Reference ranges for sMg levels in preterm infants are difficult to pinpoint because a limited number of studies evaluate the sMg concentration in healthy preterm and term infants during the first week of life. Some data have been published on the cord blood from healthy preterm and term infants and can be utilized to estimate early neonatal levels. In addition, data on sMg levels in cord blood and during the early neonatal period are available in preterm infants included in randomized control trials evaluating the effect of maternal magnesium administration before delivery for tocolysis, for prevention or treatment of eclampsia, or for neuroprotection against cerebral palsy. In these studies, the control group may represent normative neonatal levels whereas the treatment group may be evaluated to discern upper tolerable levels or potential side effects of magnesium overload (Table S2; Figure 2) [24,34,35,40,41,42,43,46,47,48,49,50,51,52,53,57,58,59,60,63,74,75,76,77,78,79,80,81].

In identified studies that sampled umbilical cord blood at delivery in preterm infants not administered magnesium during pregnancy, mean magnesium concentrations varied considerably, ranging from 0.67 to 0.96 mmol/L (Table S2) [24,34,75,76,77,78,81]. Similar mean values were observed in studies of term infants at delivery, in which the mean concentration of sMg in cord blood ranged from 0.61 to 0.97 mmol/L [35,40,41,43,46,48,50,51,52,59,60,63,75,76,77,78,79,80]. Together, these data represent a potential cord sMg mean level of 0.61–0.97 mmol/L in neonates without magnesium supplementation during pregnancy. 

Although the heterogeneity of the included studies was high (*I*^2^ = 98.3%; *p* < 0.001), the meta-analysis performed on 22 studies including 2766 infants revealed a mean estimate of 0.76 mmol/L (95% CI: 0.52, 0.99) in neonates at delivery (Figure 2). For the studies not included in the meta-analysis [42,46,77,79,81], the mean (or median) reported magnesium level falls within the derived 95% CIs, as well as the reported ranges, except for Stigson et al. (1997), which reported a wider range (possibly due to a lack of exclusion criteria relevant to medical conditions) [81].

These mean values are similar to that observed in mothers during pregnancy, failing to demonstrate a distinct relationship between gestational age and neonatal magnesium concentration. However, magnesium crosses the placental barrier actively [11,82], and magnesium levels in neonates tend to reflect maternal magnesium concentrations, regardless of magnesium exposure during pregnancy (Table 1) [24,35,40,42,47,48,49,50,51,52,53,59,60,63,79,83,84,85]. Supporting data are provided in studies conducted in term neonates without magnesium supplementation during pregnancy [42,49,50], as well as in studies conducted in both preterm and term neonates with magnesium supplementation during pregnancy [79,83,85]. The results from the study by Marret et al. (2008) also support the positive relationship between magnesium levels in cord blood and maternal blood [24]. Although the influence of other maternal factors (e.g., method of delivery and multiple gestations) have been proposed [21], their role in predicting neonatal magnesium concentrations has not been confirmed. As suggested for sMg concentration in healthy pregnant women without magnesium supplementation, the cord blood mean (and 95% CI) for total sMg concentration could differ from the normal reference interval reflecting an adequate fetal status.

### 3.3. Magnesium Levels at Birth in Neonates with Magnesium Supplementation during Pregnancy

Data from available studies of neonates receiving magnesium administration during pregnancy are summarized in Table S3, with considerable across-study variation in magnesium regimen. Magnesium levels were, not surprisingly, generally higher among infants with administered magnesium during pregnancy compared to those without supplementation, with emphasis on the extent of cumulative intake provided before delivery. Average cord blood sMg concentrations drawn at delivery ranged from 0.80 to 1.80 mmol/L in studies of magnesium supplementation for pregnancy-induced hypertension (PIH), fetal neuroprotection, or prevention of eclampsia [24,40,79,86,87,88,89].

In the study by Rudnicki et al. (1991), which reported 0.80 mmol/L at delivery in term infants of mothers with PIH, mothers received a 48-h intravenous infusion (50 mmol of MgCl_2_ during the first 24 h followed by 12 mmol of MgCl_2_ during the second 24 h), followed by 15 mmol/d until one day after delivery, with a median duration of 12 days (range: 3–23 days) [79]. This was a low-dose magnesium regimen, which may explain why the level at delivery was similar to corresponding levels seen in term infants without magnesium supplementation during pregnancy.

In two studies for preterm neuroprotection [24,87], the average magnesium concentrations were 1.00 mmol/L and 1.30 mmol/L, which seem to reflect the infant’s exposure to magnesium administration in women at risk of imminent very preterm birth. In the PREMAG study, which reported a mean sMg of 1.00 ± 0.22 mmol/L at birth (*n* = 191), mothers received MgSO_4_ as a single injection of 0.1 mg/L (4 g, 33.23 mmol) over 30 min within 24 h of expected delivery. In contrast, in the study by Palatnik et al. (2015), which reported a mean of 1.30 mmol/L at birth, mothers received at least a loading dose of 6 g (49.85 mmol) infused for 20–30 min, followed by a maintenance infusion of 2 g/h (16.62 mmol/h) for up to 12 h [87]. In this study, infusion was discontinued if delivery was no longer imminent, but would be resumed if the anticipated delivery was at less than 34 weeks gestation.

The other 2 studies reported magnesium concentrations in infants with preeclamptic mothers who received magnesium supplementation for prevention of eclampsia [40,86]. Although the severely preeclamptic mothers in the study by Katz et al. (2012) received MgSO_4_ prior to delivery, the regimen was unspecified; the resulting sMg level in venous umbilical cord blood was 1.37 mmol/L [40]. The mean sMg level in term infants whose mothers received MgSO_4_ supplementation in the study by Boriboonhirunsarn et al. (2012) was 1.80 mmol/L at delivery [86]. The magnesium regimen for these mothers included a loading dose of 4 g (33.23 mmol) of MgSO_4_ followed by continuous intravenous infusion of 2 g/h (16.62 mmol/h), with dose adjustments performed depending on if the magnesium level was within therapeutic range (2.0–3.5 mmol/L) and on clinical signs and symptoms. The total dose ranged from 5.5 g (45.69 mmol) to 34.5 g (286.62 mmol), with a mean of 14.4 g (119.63 mmol) [86].

**Figure 3:**
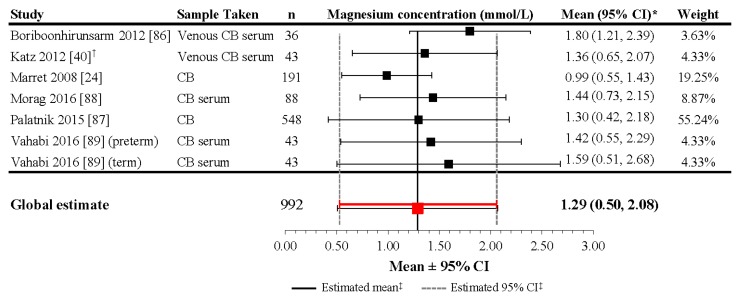
Magnesium concentrations in umbilical cord blood of newborns with magnesium supplementation during pregnancy. * If 95% CIs were not reported in the study, then the 95% CIs were calculated as follows: mean ± (1.96 × SD). ^†^ Patient groups within these studies were pooled, as the literature search did not confirm an association between magnesium levels and gestational age, birth weight, or mode of delivery. ^‡^ Reference line and 95% CI derived using only studies reporting both mean and SD data. Overall statistic for heterogeneity: *I*^2^ = 99.1% (*p* < 0.001). CB: [umbilical] cord blood; CI: confidence interval; n: number of infants; SD: standard deviation.

Although the heterogeneity of the included studies was high (*I*^2^ = 99.1%; *p* < 0.001), the meta-analysis performed on six studies including 992 preterm and term infants revealed a mean estimate of 1.29 mmol/L (95% CI: 0.50, 2.08) at delivery in neonates with exposure to magnesium supplementation at the end of pregnancy (Figure 3). The study by Rudnicki et al. (1991) was not included in the meta-analysis (as it did not report mean values in infants from healthy mothers) and reported a median sMg level of 0.80 mmol/L at delivery in term infants of mothers with PIH [79]. In comparison to the meta-analysis results, this median value is more similar to infants without magnesium supplementation during pregnancy, which may be related to the relatively low dosing regimen employed by the Rudnicki et al. (1991) study in comparison to the others, as well as the fact that the study used a different type of magnesium supplementation formula (MgCl_2_ vs. MgSO_4_).

Neonates receiving magnesium supplementation during pregnancy have higher reported concentrations of magnesium at birth than infants with no exposure, regardless of gestational age and indication for treatment. Variability in reported levels may be related to the duration of supplementation during pregnancy, the total dose of magnesium received, and the delay between last supplementation dose and delivery, as well as other heterogeneous characteristics among study populations.

### 3.4. Magnesium Levels in Neonates during the First Days of Life

Serum magnesium concentration at birth is directly related to the maternal concentration and thus directly related to the magnesium intake during pregnancy (Table 1). After birth, several factors influence the sMg concentration during the first days of life. 

#### 3.4.1. Infants without Prenatal Magnesium Supplementation

**Figure 4:**
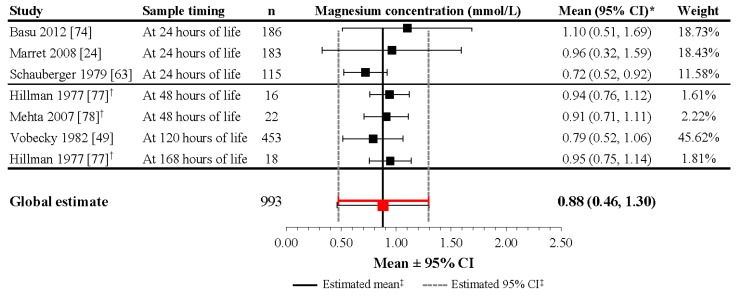
Magnesium concentrations during the first week of life in healthy newborns without magnesium supplementation during pregnancy. * If 95% CIs were not reported in the study, then the 95% CIs were calculated as follows: mean ± (1.96 × SD). ^†^ Patient groups within these studies were pooled, as the literature search did not confirm an association between magnesium levels and gestational age, birth weight, or mode of delivery. ^‡^ Reference line and 95% CI derived using only studies reporting both mean and SD data. Overall statistic for heterogeneity: *I*^2^ = 98.5% (*p* < 0.001). CI: confidence interval; n: number of infants; SD: standard deviation.

In infants that did not receive magnesium supplementation during pregnancy, the reported mean sMg levels from the 6 studies included in the meta-analysis ranged from 0.72 to 1.10 mmol/L within 24 h after delivery [24,63,74,79], from 0.94 to 0.97 mmol/L at 48 h after delivery [77,78], and from 0.79 to 0.95 mmol/L at 5–7 days of life (Table S2) [49,77]. Although the heterogeneity of the included studies was high (*I*^2^ = 99.5%; *p* < 0.001), the meta-analysis performed on 993 preterm and term infants revealed a mean estimate of 0.88 mmol/L (95% CI: 0.46, 1.30) during the first week of life in neonates without exposure to magnesium supplementation during pregnancy (Figure 4). 

Studies included in this meta-analysis of infants with no magnesium administration during pregnancy varied in demonstrating a distinct trend in the change in magnesium concentration from birth during the first days of life. In the largest study, mean magnesium concentration increased from birth (0.68 mmol/L) to Day 5 of life (0.79 mmol/L) [49]. Other studies that demonstrated an increase in magnesium levels include those by Marret et al. (2008), Schauberger et al. (1979), and Mehta et al. (2007). Marret et al. (2008) reported a significant mean increase from 0.81 mmol/L at birth to 0.96 mmol/L, (*p* = 0.001) 24 h after delivery in preterm infants [24]. For preterm and term infants evaluated in the Schauberger et al. (1979) and Mehta et al. (2007) studies, the increases in mean magnesium levels at 24 h and at 36–48 h after delivery, respectively, were very small (difference of 0.01 mmol/L for both populations) [63,78]. An interesting result was found in the Hillman et al. (1977) study, which reported an increase in mean magnesium levels from birth to 48 ± 2 h post-delivery in both preterm and term infants; however, at seven days of life, the mean sMg level for term infants decreased, while mean sMg concentration for preterm infants continued to increase [77]. Three studies found from the literature search, but which were not included in the meta-analysis, also show different variation in magnesium changes. Rudnicki et al. (1991), which was not included because it provided only a median value for infants delivered by mothers with PIH, demonstrated a decrease in median magnesium levels from birth (0.77 mmol/L) to 24 h postpartum (0.72 mmol/L) [79]. In a small study by Schanler et al. (1997), which did not provide raw data, sMg concentrations in infants not administered magnesium during pregnancy remained at approximately 1 mmol/L during the first 72 h of life, with a slight downward trend [85]. According to data from the CALIPER database, which combined data for newborns aged 0–14 days, magnesium concentrations in healthy newborns initially increased during the first days of life, then steadily decreased progressively during the second week of life to reach adult levels [16]. These discrepancies may be related to heterogeneity among the populations, including postnatal magnesium intake, renal function maturation, gestational age, and birth weight. 

The results of the meta-analyses in infants without prenatal magnesium supplementation suggest that sMg levels in these infants increase during the first week of life (mean cord blood Mg concentration at birth, 0.76 mmol/L; mean sMg concentration during first week, 0.88 mmol/L), aligning with the trend found from the large CALIPER database.

#### 3.4.2. Infants with Prenatal Magnesium Supplementation

**Figure 5:**
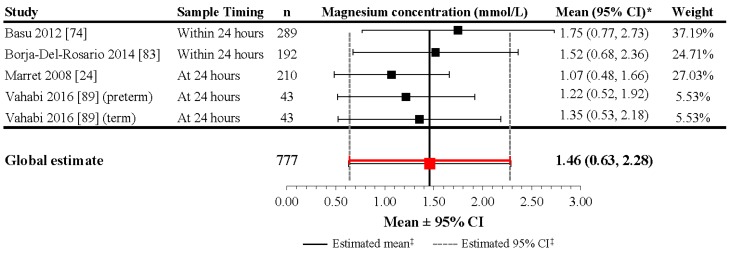
Magnesium concentrations during the first day of life in newborns with magnesium supplementation during pregnancy. * If 95% CIs were not reported in the study, then the 95% CIs were calculated as follows: mean ± (1.96 × SD). ^‡^ Reference line and 95% CI derived using only studies reporting both mean and SD data. Overall statistic for heterogeneity: *I*^2^ = 99.5% (*p* < 0.001). CI: confidence interval; n: number of infants; SD: standard deviation.

In infants exposed to magnesium supplementation during pregnancy, the reported mean sMg levels from five studies ranged from 0.89 to 1.75 mmol/L within 24 h after delivery (Table S3) [24,74,79,83,89]. Although the heterogeneity of the included studies was high (*I*^2^ = 99.5%; *p* < 0.001), the meta-analysis performed on four studies including 777 infants evaluated at 24 h revealed a mean estimate of 1.468 mmol/L (95% CI: 0.634, 2.28) at 24 h in neonates with magnesium supplementation during pregnancy (Figure 5). The lower levels reported in the Marret et al. (2008) study seem to be the result of the lower cumulative magnesium intake provided before delivery [24,79]. In the Basu et al. (2012) and Borja-Del-Rosario et al. (2014) studies, which reported high magnesium levels at 24 h, mothers received a loading dose of 4–6 g (33.23–49.85 mmol) or 6 g (49.85 mmol), respectively, infused over 30 min, followed by a maintenance infusion of 1–2 g/h (8.31–16.62 mmol/h) or 2 g/h (16.62 mmol/h), respectively, until delivery [74,83].

Similar to levels reported in infants without magnesium supplementation during pregnancy, these studies varied in demonstrating a distinct trend in the change in magnesium concentration from birth during the first days of life. The results of the meta-analyses suggest that sMg levels in infants increase during the first day of life (mean cord blood Mg concentration at birth, 1.25 mmol/L; mean sMg concentration at 24 h, 1.48 mmol/L), aligning with the results of the meta-analyses in neonates that did not receive magnesium administration during pregnancy. Two of the studies reported a small positive change in mean magnesium concentration between birth and at 24 h post-delivery [24,79]. In the study by Marret et al. (2008), sMg increases from 0.99 mmol/L at birth to 1.07 mmol/L (*p* = 0.001) 24 h after delivery [24]. In addition, both studies demonstrated larger first-day average magnesium concentration in infants with supplementation compared to those without [24,79]. In the study by Schanler et al. (1997), sMg concentrations demonstrated an overall negative trend from birth (approximately 1.75 mmol/L) to 72 h (approximately 1.46 mmol/L) [85]. In this study for tocolysis, mothers received a loading dose of 6 g (49.85 mmol) of MgSO_4_ over 30 min, followed by a maintenance dose of 2 g/h (16.62 mmol/h; titrated from 1.5 to 3 g/h (12.46–24.92 mmol/h) depending upon uterine activity) until within 60 min of birth or at the time of the C-section, with a mean administration of 26 days (range: 8–63) [85]. 

Notably, a relationship between the extent of magnesium exposure (dosage) and neonatal magnesium has been documented in two of these studies [83,85]. Borja-Del-Rosario et al. (2014) reported significant correlations between the total maternal magnesium dose at 24 and 48 h of infusion and neonatal sMg concentrations (*r* = 0.55 (*p* < 0.0001) and *r* = 0.35 (*p* < 0.0001), respectively) [83]. Similarly, Schanler et al. (1997) described a significant positive correlation between maternal MgSO_4_ dosage and neonatal sMg concentration (*r* = 0.74; *p* < 0.001) when MgSO_4_ was administered for preterm labor [85]. 

### 3.5. Factors Contributing to Magnesium Levels in Neonates during the First Days of Life

As demonstrated in the previous sections, magnesium supplementation during pregnancy is highly correlated with neonatal sMg levels. After birth, many other factors contribute to influencing neonatal sMg levels, including postnatal magnesium intake, renal function, gestational age, and birth weight. 

In preterm infants, magnesium nutritional supply can be provided parenterally during the first days of life followed by a progressive introduction of enteral nutrition as human milk or formula. In parenteral nutrition, magnesium intake is fully available for mineral metabolism and magnesium homeostasis. With enteral nutrition, the intestinal magnesium absorption is limited among newborns but may be higher in preterm versus term infants [11]. Magnesium retention may also be higher in preterm versus term infants and may exceed the apparent intrauterine retention rates [90]. In term infants, breast milk provides approximately 5.5–7.5 mg/kg/day (0.23–0.31 mmol/kg/day) of magnesium [17]. By contrast, amounts of magnesium provided by enteral or parenteral nutrition in preterm infants vary, depending on the preparation(s) administered. With fortified human milk or formula mean magnesium intakes range from 6.0 to 12.0 mg/kg/day (0.25–0.5 mmol/kg/day), absorption rate around 50%, and mean retention accounted from 1.8 to 3.6 mg/kg/day (0.08–0.15 mmol/kg/day) [11].

Magnesium levels among newborn infants, especially VLBW infants, change rapidly during the first days of life as a result of the early parenteral and enteral magnesium intakes and the maturation of the glomerular filtration rate. After a few days, sMg levels decrease progressively to reach a plateau at the end of the first month of age. Indeed, renal immaturity is observed at birth and renal function improves progressively during the first weeks of life. In VLBW infants, renal function is directly related to gestational age and inversely related to postnatal age as illustrated by the serum creatinine concentration [91]. There is a progressive maturation of the glomerular filtration rate increasing during the first weeks of life. Tubular reabsorption of magnesium is high but the serum threshold of urinary excretion is not well defined in VLBW infants [92,93]. Medications for patent ductus arteriosus (e.g., indomethacin, ibuprofen, acetylsalicylic acid) affecting renal perfusion can potentially influence magnesium handling [94,95]. Additionally, changes in hormonal and nonhormonal factors (e.g., calcium) can potentially contribute to magnesium homeostasis and sMg levels. 

Data from studies conducted in VLBW infants are often confounded by postnatal parenteral magnesium supplementation. In a time-course study of magnesium homeostasis, extremely low birth weight infants with a birth weight (<1000 g; *n =* 51) received a parenteral solution providing 0.15 mmol/kg/day of magnesium. Mean sMg increased during the first week of life (from 0.85 ± 0.14 mmol/L on Day 1 to 1.09 ± 0.10 mmol/L on Day 4), then decreased progressively (to 0.89 ± 0.08 mmol/L on Day 21 and to 0.91 ± 0.10 mmol/L on Day 28) [96]. This trend may be strongly influenced by glomerular function. During the first week, the sMg concentration ranged from 0.62 mmol/L to 1.53 mmol/L, but four out of five infants with magnesium levels higher than 1.3 mmol/L had evidence of acute kidney injury, defined as serum creatinine concentration greater than 1.5 mg/dL (132 mmol/L). In this study, higher sMg concentration appears to be related to birth weight (<750 g) and/or gestational age (<27 weeks) [96].

**Figure 6:**
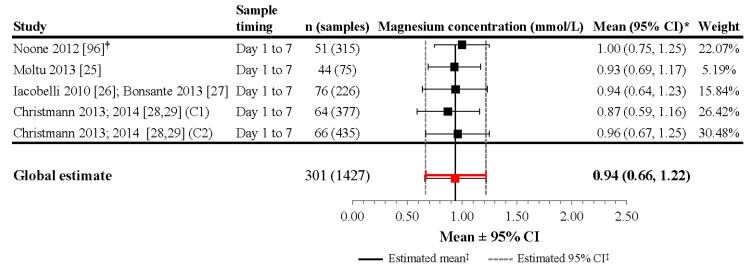
Magnesium concentrations during the first week of life in very-low-birth-weight newborns receiving parenteral solution. * If 95% CIs were not reported in the study, then the 95% CIs were calculated as follows: mean ± (1.96 × SD). ^†^ Patient groups within these studies were pooled, as the literature search did not confirm an association between magnesium levels and gestational age, birth weight, or mode of delivery. ^‡^ Reference line and 95% CI derived using only studies reporting both mean and SD data. Overall statistic for heterogeneity: *I*^2^ = 62.8% (*p* = 0.045). C1: cohort 1; C2: cohort 2; CI: confidence interval; n: number of infants; SD: standard deviation.

Additional data were obtained from nutritional intervention studies evaluating the effect of early aggressive parenteral nutrition in VLBW infants by direct contact with the primary authors (Figure 6) [25,26,27,28,29,97]. The first study was a randomized controlled trial that evaluated the effect of two diets (parenteral plus enteral) on postnatal growth and included 48 VLBW neonates (<1500 g) in two groups of 24 infants [25]. The starting parenteral solutions contained 0.23 mmol/dL and 0.20 mmol/dL of Mg for the two groups. The mean parenteral magnesium intake was 0.084 ± 0.034 mmol/kg/day and 0.093 ± 0.030 mmol/kg/day in the intervention and control group, respectively (*p* = 0.18). Mean sMg levels reported at Day 3 of life were 0.91 ± 0.13 mmol/L (*n* = 22) and 0.96 ± 0.12 mmol/L (*n* = 21), respectively. During the first week of life in the whole population, the mean sMg concentrations were 0.93 ± 0.12 mmol/L (*n* = 75) [25] (Figure 6). Prenatal magnesium exposure was not part of the data collected in this study; however, based on reviewing the magnesium data, the authors believe that although it is possible some of the infants could have received prenatal magnesium supplementation, the number of these patients is minimal and would not be expected to significantly alter the results and interpretations of the current analysis.

The second nutritional intervention study was a cohort study evaluating the effect of increasing protein intake during the first days of life in VLBW infants [27,97]. Serum magnesium concentration was included in the biological survey during the first week of life. In all, 226 sMg concentrations were recorded in 76 preterm infants <33 weeks gestational age. Parenteral nutrition was provided from birth and magnesium after the second day of life. Mean magnesium parenteral intake during the first week of life averaged of 0.11 mmol/kg/day of magnesium, with a maximum intake of 0.36 mmol/kg. Mean sMg concentration during the first week of life was 0.94 ± 0.15 mmol/L (range: 0.56–1.35) (Figure 6). In this study, the sMg levels increased progressively until day 5 and decreased thereafter. In addition, the mean sMg concentrations were significantly related to the parenteral magnesium intake [27,97]. The last study compares two successive cohorts of VLBW infants receiving different nutritional intakes. Both cohorts received parenteral nutrition, with the second cohort receiving higher concentrations of protein as well as minerals (including magnesium) [28,29]. For cohort 1, the maximum parenteral intake of magnesium was 0.14 mmol/kg/day while the maximum intake in cohort 2 was 0.3 mmol/kg/day. In preterm infants without prenatal magnesium supplementation, mean sMg concentration during the first week of life was 0.87 ± 0.15 mmol/L (*n =* 64; 377 samples) in cohort l and 0.96 ± 0.15 mmol/L (*n =* 66; 435 samples) in cohort 2 (Figure 6). In both groups, the mean sMg level increased up to the Day 4 of life; however, the sMg concentration from Day 1 to Day 7 in cohort 2, was significantly higher than that in cohort 1 in relation to the higher parenteral Mg intakes. In this study, sMg concentrations were also obtained during the first week of life in 17 infants with prenatal magnesium supplementation. Mean sMg concentration reached 1.35 ± 0.44 mmol/L (*n* = 114), but decreased progressively from birth to the end of the first week of life.

Although the heterogeneity of the included studies was significant (*I*^2^ = 62.8%, *p* = 0.045), the meta-analysis performed on four studies including 301 infants mainly without prenatal magnesium supplementation and 1427 evaluations during the first week of age revealed a mean estimate of 0.94 mmol/L (95% CI: 0.66, 1.22) in VLBW infants receiving total or partial parenteral nutrition (Figure 6). 

Data during the second and third weeks of life were obtained from the Noone et al. (2012) and Christmann et al. (2013; 2014) studies. In the study by Christmann et al. (2013; 2014), where enteral nutrition accounted for more than 50% of magnesium intake, mean sMg was similar in both cohorts. In cohort 1, sMg decreased respectively to 0.89 ± 0.12 mmol/L (*n =* 172) and 0.87 ± 0.07 mmol/L (*n =* 58) in cohort 1 and to 0.91 ± 0.10 mmol/L (*n =* 88) and 0.86 ± 0.08 mmol/L (*n =* 16) in cohort 2 [28,29]. Similarly, Noone et al. (2012) reported a mean value of 0.91 ± 0.10 mmol/L (*n =* 51) and 0.89 ± 0.08 mmol/L (*n =* 51) during the second and the third week of life respectively [96].

Thus, the results from the Noone et al. (2012) and Christmann et al. (2013; 2014) studies in VLBW infants during the first month of life support the results from the large database of Colantonio et al. (2012), which found that magnesium concentrations in healthy newborns initially increased during the first week of life, before steadily decreasing to adults levels [16].

In summary, the results of this review of magnesium levels during the first month of life in preterm infants and VLBW infants receiving nutritional intervention were similar to the data observed in term infants suggesting that sMg concentration increases during the first week of life to a level higher than the adult reference values and decreases to adult levels thereafter. These early changes are likely influenced by many factors, including postnatal magnesium intake, gestational age, and renal maturity.

### 3.6. Benefits and Risks of Elevated Neonatal Serum Magnesium

#### 3.6.1. Benefit: Neuroprotection

Magnesium supplementation via magnesium administration during pregnancy may provide neuroprotective benefits in preterm infants, including reduced rates of cerebral palsy and gross motor dysfunction [72,73,98]. A systematic review of data from five trials (6145 infants) indicated that antenatal MgSO_4_ supplementation in women at risk of very preterm birth reduced the risk of cerebral palsy by 32% (relative risk (RR): 0.68; 95% CI: 0.54, 0.87) [72]. In the 4 studies in which MgSO_4_ was administered with neuroprotective intent, the combined rate of death or cerebral palsy was significantly reduced (RR: 0.85, 95% CI: 0.74, 0.98) [72]. Both single-dose (4 g unrepeated) and multiple-dose (4–6 g loading followed by 1–2 g/h maintenance) regimens were studied; however, an ideal dosing regimen remains to be defined [99]. The target sMg concentration also remains unclear. 

Data from a small retrospective analysis of sMg levels and motor outcome conducted in 75 premature infants (<1500 g, 25.8 weeks gestation; 20% with MgSO_4_ supplementation during pregnancy) demonstrated a statistically significant relationship between lower magnesium levels (≤1.9 mg/dL) in the neonatal period (average age at first draw, 3.5 days) and abnormal motor exam findings (*p* = 0.037). In addition, infants with a lower magnesium level in the neonatal period tended to have a higher incidence of epilepsy (*p* = 0.060); however, logistic regression analysis including birth weight and magnesium concentration was non-significant (possibly due to the relative low sample size of the study) [100].

In 3 studies, short-term postnatal administration of MgSO_4_ (250 mg/kg/day for 3 days) has also demonstrated neuroprotective effects in term infants with severe perinatal asphyxia [101,102,103]. In the first study, mean sMg was 0.66 mmol/L at admission and 1.60 mmol/L (range, 0.94–2.05) after 48 h [103]. Reported mean sMg concentrations remained at or above 1.2–1.3 mmol/L during the 72-h treatment period and peaked at 2.8–3.2 mmol/L following the third MgSO_4_ dose in the two other studies [101,102]. An additional beneficial effect of magnesium supplementation has been also suggested to reduce bronchopulmonary dysplasia [104] and/or relative hypertension in children [105].

#### 3.6.2. Risk: Adverse Outcomes

MgSO_4_ supplementation during pregnancy appears to be generally safe for the neonate. In a meta-analysis of data from five trials and 6145 infants, evidence suggested that magnesium supplementation during pregnancy reduces the need for ongoing respiratory support and has no impact on other key pediatric outcomes (e.g., mortality, intraventricular hemorrhage, periventricular leukomalacia, low Apgar score, need for active resuscitation, convulsions, hypotonia, chronic lung disease, and postnatal corticosteroid use) [72]. More recently, in their retrospective cohort study of cardiorespiratory effects of magnesium exposure during pregnancy in preterm neonates (23–28 weeks gestation), DeJesus et al. (2015) reported similar risk of intubation in the delivery room, respiratory support during the first day of life, patent ductus arteriosus, and other neonatal outcomes in newborns with MgSO_4_ supplementation during pregnancy (*n =* 1091) relative to those without exposure (*n =* 453), as well as lower risk of later (Day 3) mechanical ventilation and hypotension in newborns with MgSO_4_ supplementation during pregnancy [106]. Indeed, ionized magnesium concentration was inversely related to umbilical artery pH that could be a confounding factor. Indeed, ionized Ca and ionized Mg in blood are ionized about 50%, but they are susceptible to the effect of blood pH, with ionization increasing when blood is on the acid side and decreasing when it is on the alkaline side [106].

Negative neonatal outcomes have been reported with elevated neonatal ionized magnesium concentrations [107]. However, ionized magnesium concentration is poorly related to total Mg content and is more related to acidosis that could be the primary factor of the outcome [108]. 

Immediate clinical outcomes in preterm infants following magnesium supplementation during pregnancy were retrospectively evaluated in 475 neonates born between 24 and 32 weeks of gestation [74]. Serum magnesium level in the first 24 h of life was used to stratify the neonates treated with magnesium during pregnancy into four subgroups: A (<1.25 mmol/L), B (1.25 to ≤1.75 mmol/L), C (1.75 to ≤2.25 mmol/L), and D (>2.25 mmol/L). In this study, magnesium during pregnancy was safe in the immediate postnatal period; however, in the subset of preterm neonates with sMg levels >2.25 mmol/L, an increased mortality independent of birth weight and gestational age was observed compared to the subgroup <1.25 mmol/L [74].

Similarly, a retrospective study at a single institution reviewed data from infants born to 6654 women who received MgSO_4_ during pregnancy for hypertensive disorders during labor and early postpartum—6 g loading dose over 20 min, followed by 2 g/h adapted to reach a therapeutic range of 2.0–3.5 mmol/L. For all infants, the delay between birth and the last maternal blood sampling was less than 4 h. Just before delivery, 30% of the mothers had a sMg concentration ranging between 1.5 and 2.0 mmol/L, and 65% had a sMg concentration ranging from 2.0 to 4.6 mmol/L [71]. In this study, mechanical ventilation in the nursery, intraventricular hemorrhage, and neonatal death were not significantly associated with maternal sMg concentrations. In contrast, 1- and 5-min Apgar scores, intubation in the delivery room, admission to special care nursery, and hypotonia were significantly increased as maternal sMg concentrations increased over 2.5 mmol/L [71]. Considering that cord blood magnesium levels are directly related to maternal levels, this large study suggests that an sMg concentration up to 2.5 mmol/L could be safe in preterm infants. 

In a study conducted in late-preterm and term infants (≥35 weeks gestation), Greenberg and colleagues (2011) demonstrated a significant relationship between total hours and total maternal dose of magnesium and admission to the neonatal intensive care unit (NICU) [109]. In this study, sMg concentration was not determined in infants not admitted in the NICU; however in the NICU, 36 of 52 neonates (69.2%) had peak sMg levels of 2.05 ± 0.75 mmol/L suggesting that late-preterm with relative hypermagnesemia were more likely to require respiratory assistance (47.8% versus 32.1%, respectively; *p* = 0.4) and/or intravenous fluid supplementation (91.3% versus 39.3%; *p* < 0.001).

Recently, an additional retrospective cohort study was developed from the Canadian Neonatal Network [110]. Resuscitation requirements and neonatal outcomes were compared between preterm infants (23–31 weeks gestation) exposed to intrapartum MgSO_4_ for neuroprotection (*n =* 1387), exposed for combined indications (neuroprotection, preeclampsia, tocolysis, and unknown) (*n =* 2147), and unexposed (*n =* 3868) [110]. This study confirmed that exposure to intrapartum MgSO_4_ is not associated with an increased need for intensive delivery room resuscitation in preterm infants under current delivery room care practices. Indeed, any resuscitation needed and mortality were significantly reduced in the exposed for neuroprotection and combined indication groups. However, after adjustment for confounding factors, only death rate remained significantly reduced (adjusted odds ratio: 0.61 (range: 0.40–0.94) and 0.64 (range: 0.46–0.89), respectively) [110].

It has also be suggested that MgSO_4_ administration during pregnancy could interfere with the closure of the ductus arteriosus [111]. In a retrospective study in 160 preterm infants (<28 weeks gestation) MgSO_4_ administration during pregnancy significantly reduced the closure of the ductus arteriosus following prophylactic indomethacin at birth compared to the control group; however, the efficacy of indomethacin treatment was similar in the two groups [111]. Moreover no differences have been observed in randomized trials using antenatal MgSO_4_ administrations for neuroprotection (PREMAG study). The threshold level of sMg concentration significantly impairing the spontaneous closure of the patent ductus arteriosus remains to be determined. 

Limited reports in preterm and term infants are available to evaluate the main clinical side effects of hypermagnesemia. Ali and colleagues (2003) described two cases of acute hypermagnesemia in premature infants resulting from malfunction of an automated total parenteral nutrition mixing device [112]. Observed effects included hypotension, QT interval prolongation, intraventricular conduction delay, respiratory depression, neuromuscular blockade, and coma [112]. In both infants, sMg levels upon admission were extremely high (21.6 mmol/L and 22.5 mmol/L, respectively) [112]. Similar effects (e.g., severe hypotension, lethargy, hypotonia, diminished deep tendon reflexes, respiratory depression, apnea, and sluggish pupillary reflexes) have been described in premature neonates with hypermagnesemia (17.8–18.4 mmol/L) of unknown etiology and no history of magnesium supplementation [113,114]. In 2013, the FDA provided a safety announcement recommending against prolonged use of MgSO_4_ to stop preterm labor as administration of MgSO_4_ injection to pregnant women longer than 5–7 days can cause low calcium levels and bone problems in the developing fetus or neonate, including thin bones (i.e., osteopenia) and bone breaks (i.e., fractures) [115].

Overall, these data suggest that neonates can tolerate during a short time a relatively wide range of sMg concentrations, without increased risk of adverse outcomes primarily associated with substantially increased sMg.

### 3.7. Implications for Postnatal Magnesium Supplementation in Preterm Infants

Clinical practice guidelines for the feeding of newborns have been regularly revised over recent decades, reflecting our improved understanding of nutrient requirements early in life [17,116,117,118,119,120,121,122,123,124,125]. Infants fed by the enteral route require higher levels of magnesium supplementation, because only approximately 30% to 50% of ingested magnesium is absorbed [11]. Taking this into consideration, the European Society for Paediatric Gastroenterology, Hepatology, and Nutrition (ESPGHAN) Committee on Nutrition in 2010 and an international group of experts in 2014 recommended that VLBW infants receiving enteral nutrition target a magnesium intake of 0.33–0.62 mmol/kg/day (8–15 mg/kg/day) [17,118]. Previous guidelines for enteral nutrition recommended that magnesium in enteral feedings could be increased as the preterm infant matured (from 0.20–0.25 mmol/kg/day for the first seven days to 0.20–0.40 mmol/kg/day from stabilization to term and 0.20–0.60 mmol/kg/day from term to one year) [119,120]. Both noted that required magnesium supplementation levels may vary depending on the feeding of other minerals that could influence the bioavailability of magnesium [119,120]. Considering a mean absorption rate around 50% [126], metabolizable magnesium can be estimated as 0.1–0.3 mmol/kg/day that could be used for adequate intakes in parenteral nutrition.

Recommendations for parenteral magnesium intake in preterm infants are limited and vary considerably; however, most propose the use of lower parenteral magnesium doses relative to those used in enteral supplementation. The American Society for Parenteral and Enteral Nutrition has recommended 0.15–0.25 mmol/kg/day in both preterm neonates and in infants and children weighing up to 50 kg [116,117]. ESPGHAN guidelines (2005) do not provide recommendations for parenteral magnesium intake in preterm infants but suggest supplementation at a rate of 0.2 mmol/kg/day for infants 0–6 months of age [123]. In a more recent clinical nutrition webinar series (in 2013), higher targets were identified for neonates weighing <2 kg (0.175–0.3 mmol/kg/day) than for neonates weighing >2 kg (0.125–0.25 mmol/kg/day) [127,128]. Recent recommendations put forth by the Chinese Society of Parenteral and Enteral Nutrition are slightly higher (0.3–0.4 mmol/kg/day in premature infants and 0.4–0.5 mmol/kg/day in term infants) [121]. However, a ready-to-use parenteral solution providing 0.38 mmol/kg/day was recently retrieved from the market in 2013 due to a risk of hypermagnesemia and was replaced by the same solution providing a maximum of 0.21 mmol/kg/day [129]. Therefore, for VLBW infants, a reduction of the magnesium intake has been suggested during the first day of life; thus, Mimouni and colleagues (2014) recommend 0–0.12 mmol/kg/day on the first day of life and 0.3–0.4 mmol/kg/day thereafter [17].

According to the data available on parenteral nutrition in preterm infants, as presented before, magnesium supplies may be provided from the first day of life in preterm infants and progressively increased during the first week in relation to the protein (amino acid) and the energy intakes. In the range of the actual recommendations (0.10–0.40 mmol/kg/day), safe levels of sMg concentration are observed. Nevertheless, in light of the apparent variability in magnesium levels reported in the preterm population, regular serum monitoring should be used to tailor supplementation for the individual patient. This is of particular importance in infants with a history of magnesium supplementation during pregnancy, infants receiving indomethacin or ibuprofen for patent ductus arteriosus prevention or treatment, and infants with transitory renal failure.

### 3.8. Limitations

These meta-analyses were limited by high levels of heterogeneity and a limited number of available studies reporting sMg concentrations in mothers and neonates. Although heterogeneity was not explored statistically, reasons for this variance include differences in magnesium intake during pregnancy, postnatal magnesium intake, number of samples taken, sample timing, gestational age, lack of standardized analytical methods to determine magnesium level, and exclusion/inclusion criteria relevant to medical conditions (e.g., hypertension, preeclampsia, diabetes, and AIDS).

## 4. Summary and Recommendations

**Figure 7:**
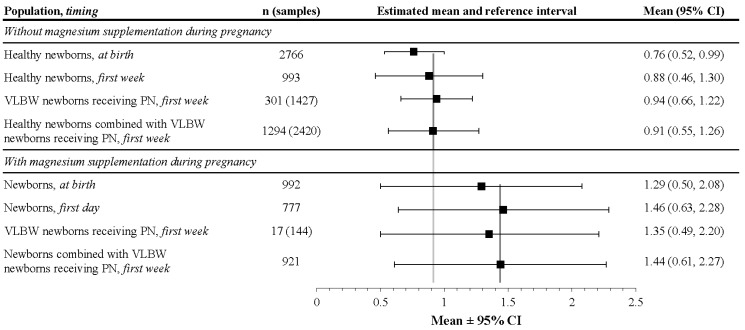
Summary of meta-analysis results of magnesium concentrations in newborns (mmol/L). CI: confidence interval; n: number of infants; PN: parenteral nutrition; VLBW: very low birth weight.

Serum magnesium levels vary broadly in preterm infants and are influenced not only by maternal factors, but also by health status (e.g., renal function), concomitant nutrient intake, and drug administration. Available data suggest that normal mean sMg levels in preterm infants during the first week of life was higher than that in the cord blood and could be estimated as 0.88 mmol/L with a reference interval ranging from 0.46 to 1.30 mmol/L (Figure 7). In infants with magnesium supplementation during pregnancy, as well as in preterm infants with relative renal failure, sMg concentrations up to 2.0 mmol/L appear to be well tolerated, requiring adequate survey and minimal intervention. It is notable that these data were fairly consistent, despite the lack of standardized analytical methods of magnesium level determination. The considerable variance in the recommendations for enteral and parenteral magnesium intake in preterm infants reflects the limited knowledge of magnesium requirements in this population; thus, regular serum monitoring is crucial to maintaining adequate magnesium levels in the individual patient. In addition, due to the demonstrated neuroprotective effect with antenatal administration of MgSO_4_, it is proposed to conduct a randomized clinical trial evaluating different dose regimens of magnesium in parenteral nutrition on neurodevelopmental outcomes and potential side effects in VLBW infants.

## Figures and Tables

**Table 1:** Studies reporting relationship between maternal and umbilical cord blood and/or serum magnesium concentrations in newborns without and with magnesium supplementation during pregnancy.

Study	Population (*n*)	Sampling Method	Maternal Magnesium Level, Mean ± SD, mmol/L	Neonatal Magnesium Level, Mean ± SD, mmol/L	Correlation
No magnesium supplementation					
Kozielec 2004 [50]	Term infants (38–41 weeks (72)) and mothers (75) derived from population of mothers (83) and neonates	At delivery, 2 mL maternal venous blood and 5 mL cord blood	0.89 ± 0.13	0.97 ± 0.16	*r* = 0.52 *p* = 0.000006
Marret 2008 [24]	Preterm infants (23–32 weeks (92)) and mothers (92)	At delivery; mothers blood serum and venous cord blood	0.78 ± 0.11	0.83 ± 0.13	*r* = 0.36 *p* = 0.0004
Schulpis 2010 [48]	Term infants and mothers - Vaginal delivery (16) - Scheduled cesarean delivery (14)	Mothers at the beginning of delivery; cord blood within 3–4 min of delivery	0.81 ± 0.09 0.81 ± 0.04	0.81 ± 0.04 0.80 ± 0.02	*r* = not provided *p* > 0.05 *r* = not provided *p* > 0.05
Vobecky 1982 [49]	Mothers (550) and healthy term infants (505)	Maternal venous blood obtained during delivery; mixed venous-arterial cord blood at delivery	0.68 ± 0.13	0.69 ± 0.13	*r* = 0.64 *p* < 0.01
Magnesium supplementation					
Borja-Del-Rosario 2014 [83]	Preterm infants 24–32 weeks (192) and their mothers	Maternal serum within 6 h before delivery; neonatal serum 24 h post-delivery	2.30 ± 0.51	1.52 ± 0.43	*r* = 0.10 *p* = 0.15
Marret 2008 [24]	Preterm infants (23–32 weeks (119)) and mothers (119)	At delivery; mothers blood serum and venous cord blood	0.77 ± 0.129	1.0 ± 0.193	*r* = 0.23 *p* = 0.012
Rudnicki 1991 [79]	Women (12) with pregnancy-induced hypertension and their infants (gestational age, 37–39 weeks)	Mothers prior to delivery; arterial blood from cord	0.74 (0.71, 0.81) ^1^	0.80 (0.70, 0.91) ^1^	*r* = 0.77 *p* < 0.02
Schanler 1997 [85]	Women in preterm labor (16) and their infants (22); mean gestational age, 31.7 ± 2.6 weeks	At delivery; peripheral venous blood from mothers and infants	Not provided (figure only)	Not provided (figure only)	*r* = 0.61 *p* = 0.004

^1^ Median (95% confidence interval). Magnesium concentrations reported in non-SI units have been converted to mmol/L as follows: mg/dL × 0.411; mEq/L × 0.5.

## Supplementary Materials

The following are available online at www.mdpi.com/2072-6643/9/10/1125/s1, Table S1: Studies reporting magnesium levels in healthy pregnant women with no magnesium supplementation during pregnancy, Table S2: Studies reporting magnesium levels in newborns with no magnesium supplementation during pregnancy, Table S3: Studies reporting magnesium levels in newborns with magnesium supplementation during pregnancy.
