# Supplementary material for: Serum Magnesium Levels in Preterm Infants Are Higher Than Adult Levels: A Systematic Literature Review and Meta-Analysis
Source: Nutrients. 2017 Oct 16;9(10):1125. doi: 10.3390/nu9101125 (PMC5691741; doi:10.3390/nu9101125)
Supplement: Supplementary file 1 [file nutrients-09-01125-s001.zip › nutrients-220828-supplementary.pdf]

Table S1. Studies reporting magnesium levels in healthy pregnant women with no magnesium supplementation during pregnancy

Study	Population (*n*)	Gestation, weeks (range or mean ±SD)	Sample and Timing	Reported Magnesium Level, mean±SD, mmol/L	Range, mmol/L
**Adam 2001 [31]**	Healthy pregnant women (20)	35–40	Venous blood plasma; Before onset of labor	0.70±0.06	–
**Adekanle 2014 [32]**	Healthy pregnant women (75)	20–42	Blood serum; At recruitment (during gestation)	0.73±0.14	0.40–1.10
**Al-Jameil 2017 [54]**	Healthy pregnant women (40)	31.17±5.33	Blood serum; At recruitment (during gestation)	0.80±0.14	–
Apostol 2010 [33]	Healthy pregnant women (16)	Unspecified	Blood serum; Unspecified (during gestation)	0.59±0.04 *	–
**Asemi 2015 [55]**	Healthy pregnant women (35)	24–28	Blood serum; At recruitment (during gestation)	0.67 (0.14)	–
**Borekci 2009 [34]**	Healthy pregnant women (16)	33.82±1.50	Blood serum; After overnight fast (during gestation)	0.76±0.02	0.74–0.78
**Cruikshank 1979 [35]**	Healthy pregnant women (11)	38.3	Venous blood serum; 2–12 h (mean, 8) before delivery	0.68±0.05	–
	Healthy pregnant women (11)	38.3	Venous blood serum; At delivery	0.54±0.05	–
**Enaruna 2013 [36]**	Healthy pregnant women (160)	24–26	Venous blood serum; At recruitment (during gestation)	0.73±0.10	–
	Healthy pregnant women (160)	>34	Venous blood serum; At delivery	0.69±0.23	–
**Hallak 1993 [37]**	Healthy pregnant women (21)	28.2±6.5	Blood serum; Unspecified (during gestation)	0.73±0.08	–
**Handwerker 1993 [46]**	Healthy pregnant women (26)	37.5–42	Venous blood serum; During labor	0.78±0.02 *	–
**Jafrin 2014 [56]**	Healthy pregnant women (42)	≥28 weeks	Blood serum; Unspecified (during gestation)	0.79±0.33	–
**Jain 2010 [38]**	Healthy pregnant women (50)	33.62±7.83	Venous blood serum; Unspecified (during gestation)	0.79±0.13	–
**Kanagal 2014 [39]**	Healthy pregnant women (60)	38.21±0.85	Venous blood serum; After overnight fast (during gestation)	0.65±0.30	–
**Katz 2012 [40]**	Healthy pregnant women (80)	38.15±2.19	Venous blood serum; Before delivery	0.62±0.15	–
**Khoushabi 2016 [57]**	Healthy pregnant women (60)	Third trimester (unspecified)	Blood serum; Third trimester (during gestation)	0.78±0.16	–
**Kocylowski 2017 [58]**	Healthy pregnant women (64)	39.2±1.3	Blood serum; Before delivery	0.73±0.06	–
**Kozielec 2004 [50]**	Healthy pregnant women (75)	38–41	Venous blood serum; Immediately after delivery	0.89±0.13	0.53–1.18

**Krachler 1999 [59]**	Healthy pregnant women (29)	Mature (unspecified)	Venous blood serum; Within 20 min of delivery	0.69±0.09	0.54–0.94
**Lazer 2012 [41]**	Healthy pregnant women (active labor; 40)	38±3	Venous blood plasma; Before onset of labor	0.54±0.08	–
	Healthy pregnant women (elective C-section; 40)	37±9	Venous blood plasma; Before onset of labor	0.66±0.17	–
**Lukacsi 1991 [47]**	Healthy pregnant women (38)	Unspecified	Umbilical cord arterial blood serum; During labor	0.77±0.07	–
**Makinde 1991 [60]**	Healthy pregnant women (33)	Term (unspecified)	Blood serum; Within 5 min of delivery	0.58±0.09	–
**Marret 2008 [24]**	Healthy pregnant women (176)	23–32	Blood serum; At delivery	0.72±0.15	–
**McGuinness 1980 [51]**	Healthy pregnant women (14)	≥37	Venous blood serum; At delivery	0.69±0.03	–
**Nabouli 2016 [61]**	Healthy pregnant women (80)	24–28	Venous blood serum; At recruitment (during gestation)	0.92±0.2	–
Newman 1957 [42]	Healthy pregnant women (27)	Third trimester (unspecified)	Blood serum; Third trimester (during gestation)	0.74	0.52–0.87
	Healthy pregnant women (27)	Unspecified	Blood serum; At delivery	0.71	0.38–0.87
**Nishiyama 1985 [52]**	Healthy pregnant women (28)	37–41	Venous blood serum; At delivery	0.79±0.08	–
**Owusu Darkwa 2017 [62]**	Healthy pregnant women (30)	>30	Venous blood serum; At recruitment (during gestation)	0.76±0.14	–
**Schauberger 1979 [63]**	Healthy pregnant women (115)	36–42	Venous blood serum; Within 2 min of delivery	0.66±0.19	–
**Schulpis 2010 [48]**	Healthy pregnant women (16)	37–41 (vaginal delivery)	Blood serum; Beginning of labor	0.81±0.09	0.54–0.95
	Healthy pregnant women (14)	37–41 (C-section)	Blood serum; Beginning of labor	0.81±0.04	0.74–0.90
	Healthy pregnant women (16)	37–41 (vaginal delivery)	Blood serum; At end of delivery	0.69±0.03	0.66–0.74
	Healthy pregnant women (14)	37–41 (C-section)	Blood serum; At end of delivery	0.80±0.02	0.74–0.82
**Sun 1990 [53]**	Healthy pregnant women (80)	Unspecified	Venous blood serum; At delivery	0.86±0.21	–
	Healthy pregnant women (63)	Unspecified	Venous blood serum; At delivery	0.90±0.16	–
**Tabrizi 2014 [43]**	Healthy pregnant women (162)	Third trimester (unspecified)	Venous blood serum; End of third trimester (during gestation)	0.86±0.12	–
**Tavana 2013 [44]**	Healthy pregnant women (52)	18–22	Venous blood serum; Unspecified (during gestation)	0.95±0.18	–
	Healthy pregnant	30–35	Venous blood serum; Unspecified (during	0.90±0.26	–

	women (52)		gestation)		
Uludag 2014 [45]	Healthy pregnant women (100)	28–33	Blood serum; Unspecified (during gestation)	0.74 †	0.62–0.86
**Vobecky 1982 [49]**	Healthy pregnant women (550)	Term (unspecified)	Venous blood serum; During labor	0.68±0.13	–

* standard error of the mean; † median. SD: standard deviation. Bold indicates inclusion in the meta-analysis. Magnesium concentrations reported in non-SI units have been converted to mmol/L as follows: mg/dL × 0.411; mEq/L × 0.5

Table S2. Studies reporting magnesium levels in newborns with no magnesium supplementation during pregnancy

Study	Population, gestational age (range or mean±SD) (*n*)	Sample and Timing	Reported Magnesium Level, mean±SD, mmol/L	Range, mmol/L
		Umbilical cord blood (CB)		
**Borekci 2009 [34]**	Preterm infants, 33.82±1.50 weeks (16)	CB; At delivery	0.83±0.34	–
**Cruikshank 1979 [35]**	Term infants, mean 38.3 weeks (8)	CB arterial serum; At delivery	0.76±0.09	–
	Term infants, mean 38.3 weeks (11)	CB venous serum; At delivery	0.75±0.09	–
**Elizabeth 2008 [75]**	Preterm infants, <37 weeks; <2.5 kg (59)	CB; Within 5 min of delivery	0.67±0.13	0.41–0.90
	Term infants, >37 weeks; <2.5 kg (192)	CB; Within 5 min of delivery	0.71±0.09	0.41–0.90
	Term infants, >37 weeks; >2.5kg (247)	CB; Within 5 min of delivery	0.81±0.08	0.49–0.99
**Fenton 2011 [76]**	Preterm infants, 23–27 weeks (48)	CB serum; At delivery	0.79±0.07	0.65–0.94 ‡
	Preterm infants, 28–31 weeks (127)	CB serum; At delivery	0.79±0.10	0.58–0.99 ‡
	Preterm infants, 32–34 weeks (193)	CB serum; At delivery	0.76±0.08	0.60–0.92 ‡
	Preterm infants, 35–36 weeks (120)	CB serum; At delivery	0.76±0.09	0.58–0.93 ‡
	Term infants, >36 weeks (53)	CB serum; At delivery	0.77±0.08	0.63–0.92 ‡
Handwerker 1993 [46]	Term infants, 37.5–42 weeks (23)	CB venous serum; At delivery	0.76±0.02 *	0.70–0.74 ‡
	Term infants, 37.5–42 weeks (38)	CB venous serum; At delivery	0.72±0.01 *	0.70–0.78 ‡
Hillman 1977 [77]	Preterm infants, 31–36 weeks (9)	CB serum; At delivery	0.81±0.07 †	0.62–1.33
	Term infants, >36 weeks (9)	CB serum; At delivery	0.78±0.02 †	0.72–0.89
**Katz 2012 [40]**	Term infants, 38.15±2.19 weeks (80)	CB arterial serum; At delivery	0.67±0.12	–
	Term infants, 38.15±2.19 weeks (80)	CB venous serum; At delivery	0.64±0.12	–
**Khoushabi 2016 [57]**	Unspecified (60)	CB serum; Before delivery (unspecified)	0.78±0.16	–
**Kocylowski 2017 [58]**	Infants, ≥34 weeks (64)	CB venous serum; Immediately after delivery	0.73±0.07	–
**Kozielec 2004 [50]**	Term infants, 38–41 weeks (72)	CB venous serum; Immediately after delivery	0.97±0.16	0.53–1.46
**Krachler 1999 [59]**	Mature infants, unspecified (29)	CB serum; At delivery	0.71±0.09	–
**Lazer 2012 [41]**	Late preterm and term infants, 38±3 weeks (active labor; 40)	CB arterial plasma; Immediately after delivery	0.72±0.10	–
	Late preterm and term infants, 38±3 weeks (active labor; 40)	CB venous plasma; Immediately after delivery	0.62±0.09	–
	Infants, 37±9 weeks (elective C-section; 40)	CB arterial plasma; Immediately after delivery	0.61±0.11	–
	Infants, 37±9 weeks (elective C-section; 40)	CB venous plasma; Immediately after delivery	0.67±0.14	–
**Lukacsi 1991 [47]**	Unspecified (38)	CB arterial serum; During labor	0.81±0.03	–
	Unspecified (38)	CB venous serum; During labor	0.85±0.05	–
**Makinde 1991 [60]**	Term infants, unspecified (33)	CB serum; At delivery of placenta	0.62±0.06	–

**Marret 2008 [24]**	Preterm infants, 23–32 weeks (157)	CB venous; At delivery	0.80 §	0.34–2.82
**McGuinness 1980 [51]**	Term infants, ≥37 weeks (14)	CB arterial serum; At delivery of placenta	0.74±0.04	–
	Term infants, ≥37 weeks (14)	CB venous serum; At delivery of placenta	0.74±0.03	–
**Mehta 2007 [78]**	Preterm infants, <32 weeks (7)	CB arterial blood; At delivery	0.96±0.12	–
	Preterm infants, 32–34 weeks (8)	CB arterial blood; At delivery	0.88±0.11	–
	Late preterm and term infants, >35 weeks (7)	CB arterial blood; At delivery	0.85±0.09	–
Newman 1957 [42]	Unspecified (21)	CB serum; At delivery	0.78	0.64–0.84
**Nishiyama 1985 [52]**	Term infants, 37–41 weeks (26)	Mixed CB venous-arterial blood; At delivery	0.82±0.11	–
Rudnicki 1991 [79]	Term infants, 37–39 weeks (13)	CB arterial blood; At delivery	0.77 §	0.65–0.85 ‡
**Schauberger 1979 [63]**	Term infants, 36–42 weeks (79)	CB arterial serum; At delivery	0.70±0.07	–
	Term infants, 36–42 weeks (115)	CB venous serum; At delivery	0.71±0.19	–
**Schulpis 2010 [48]**	Term infants, 37–41 weeks, vaginal delivery (16)	CB serum; Within 3-4 min of delivery	0.81±0.04	0.78–0.86
	Term infants, 37–41 weeks, scheduled C-section (14)	CB serum; Within 3-4 min of delivery	0.80±0.02	0.77–0.82
**Speich 1992 [80]**	Term infants, 39.5±1.39 weeks (66)	CB venous plasma; At delivery	0.85±0.08	–
Stigson 1997 [81]	Preterm infants, 24–32 weeks (59)	CB serum; At delivery	0.81	0.32–1.28
**Sun 1990 [53]**	Unspecified (80)	CB venous serum; At delivery	0.90±0.24	–
	Unspecified (63)	CB venous serum; At delivery	0.78±0.11	–
**Tabrizi 2014 [43]**	Infants, third trimester (162)	CB serum; Before delivery of placenta	0.82±0.11	–
**Vobecky 1992 [49]**	Term infants, unspecified (505)	CB mixed venous-arterial blood; At delivery	0.68±0.13	–
		**Serum magnesium levels during the first days of life**		
**Basu 2012 [74]**	Preterm infants, 22–32 weeks (186)	Serum within 24 hours after delivery	1.10±0.3	–
Hillman 1977 [77]	Preterm infants, 31–36 weeks (8)	Blood serum by heel stick; At 48±2 hours after delivery	0.94±0.04 †	0.77–1.10
	Term infants, >36 weeks, C-section (8)	Blood serum by heel stick; At 48±2 hours after delivery	0.94±0.03 †	0.78–1.08
	Preterm infants, 31–36 weeks (9)	Blood serum by heel stick; At 7 days after delivery	0.99±0.04 †	0.71–1.13
	Term infants, >36 weeks, C-section (9)	Blood serum by heel stick; At 7 days after delivery	0.90±0.03 †	0.76–1.05
**Marret 2008 [24]**	Preterm infants, 23–32 weeks (157)	Blood by venipuncture; At 24 hours after delivery	0.85 §	0.40–2.50
**Mehta 2007 [78]**	Preterm infants, <32 weeks (7)	Blood; At 36–48 hours after delivery	0.97±0.12	–
	Preterm infants, 32–34 weeks (8)	Blood; At 36–48 hours after delivery	0.89±0.11	–
	Term infants, >35 weeks (7)	Blood; At 36–48 hours after delivery	0.87±0.09	–
Rudnicki 1991 [79]	Term infants, 37–39 weeks (13)	Blood serum by heel stick; At 24 hours after delivery	0.72 §	0.65–0.86 ‡
**Schauberger 1979 [63]**	Term infants, 36–42 weeks (115)	Blood serum by heel stick; At 24 hours after delivery	0.72±0.10	–
**Vobecky 1982 [49]**	Term infants, unspecified (453)	Capillary blood; At day 5 of life	0.79±0.14	–

* standard error of the mean; † standard error; ‡ 95% confidence interval; § median; ¦¦ number of samples. C-section: cesarean delivery; SD: standard deviation. Bold indicates inclusion in the meta-analysis. Magnesium concentrations reported in non-SI units have been converted to mmol/L as follows: mg/dL × 0.411; mEq/L × 0.5

Table S3. Studies reporting magnesium levels in newborns with magnesium supplementation during pregnancy

Study	Population, gestation (range or mean±SD) (*n*); indication for treatment	Magnesium Dosage	Sample and Timing	Reported Magnesium Level, mean±SD, mmol/L	Range, mmol/L
		**Umbilical cord blood (CB)**			
**Boriboonhirunsarn 2012 [86]**	Infants, 32–41 weeks (36); Mothers with preeclampsia	Loading dose of 4 g of $MgSO_4$ followed by continuous intravenous infusion of 2 g/h (total dose ranged from 5.5–34.5 g, mean 14.4 g)	CB venous serum; Immediately after delivery	1.80±0.30	1.40–2.90
**Katz 2012 [40]**	Late preterm and term infants, 37.72±2.56 weeks (43); Mothers with severe preeclampsia	Received magnesium sulfate [$MgSO_4$], unspecified regimen	CB venous serum; At delivery	1.36±0.36	–
	Late preterm and term infants, 37.72±2.56 weeks (43); Mothers with severe preeclampsia	Received magnesium sulfate [$MgSO_4$], unspecified regimen	CB arterial serum; At delivery	1.37±0.35	–
**Marret 2008 [24]**	Preterm infants, 23–32 weeks (88); Preterm neuroprotection	One-time administration of 4 g of $MgSO_4$ infused over 2030 min	CB; At delivery	0.99 *	0.21–2.89
**Morag 2016 [88]**	Preterm infants, <32 weeks (157); Preterm neuroprotection or preeclampsia	Intravenous loading dose of 5 g of $MgSO_4$ over 30 min, followed by maintenance infusion of 2 g/h	CB serum;	1.44±0.36	0.66–2.34
**Palatnik 2015 [87]**	Preterm infants, 30.1±2.9 weeks (548); Preterm neuroprotection	Loading dose of 6 g infused for 20–30 min, followed by maintenance infusion of 2 g/h for ≤12 hours. Infusion was discontinued if delivery was not imminent, with the intent of resuming it again for recurrent preterm labor or anticipated delivery <34 weeks gestation. Another loading dose was administered at resumption if ≥6 h passed since $MgSO_4$ discontinuation	CB; At delivery	1.30±0.45	–
Rudnicki 1991 [79]	Term infants, 37–39 weeks (12); Mothers with pregnancy-induced hypertension	48-hour intravenous infusion of $MgC1_2$: 50 mmol during the first 24 h followed by 12 mmol during the second 24 h. Starting day 3, received 15 mmol each day until 1 day after delivery	CB arterial; At delivery	0.80 *	0.70–0.91 †
**Vahabi 2016 [89]**	Preterm infants, 20–37 weeks (43); Mothers with preeclampsia	Primary 15 g injection of $MgSO_4$ (10 g intramuscularly; 5 g intravenously), followed by maintenance dose of 5 g intramuscularly every 6 h	CB serum; Immediately after delivery	1.59±0.55	–

	Term infants, ≥38 weeks (43); Mothers with preeclampsia	Primary 15 g injection of $MgSO_4$ (10 g intramuscularly; 5 g intravenously), followed by maintenance dose of 5 g intramuscularly every 6 h	CB serum; Immediately after delivery	1.42±0.45	–
		**Serum magnesium levels during the first days of life**			
**Basu 2012 [74]**	Preterm infants, 24–32 weeks (289); Preterm neuroprotection	Loading dose of 6 g infused over 30 min, followed by a maintenance infusion of 2 g/h until time of delivery	Blood serum; Within 24 hours after delivery	1.75±0.5	–
**Borja-Del-Rosario 2014 [83]**	Preterm infants, 24–32 weeks (192); Preterm neuroprotection	Loading dose of 4–6 g infused over 30 min, followed by a maintenance infusion of 1–2 g/h	Blood serum; Within 24 hours after delivery	1.52±0.43	–
**Marret 2008 [24]**	Preterm infants, 23–32 weeks (157); Preterm neuroprotection	One-time administration of 4 g of $MgSO_4$ infused over 20–30 minutes	Blood by venipuncture; At 24 hours after delivery	0.98 *	0.71–2.52
Rudnicki 1991 [79]	Term infants, 37–39 weeks (12); Mothers with pregnancy-induced hypertension	48-hour intravenous infusion of $MgCl_2$: 50 mmol during the first 24 h followed by 12 mmol during the second 24 h. Starting day 3, received 15 mmol each day until 1 day after delivery	Blood serum by heel stick; At 24 hours after delivery	0.89 *	0.74–1.01 †
**Vahabi 2016 [89]**	Preterm infants, 20–37 weeks (43); Mothers with preeclampsia	Primary 15 g injection of $MgSO_4$ (10 g intramuscularly; 5 g intravenously), followed by maintenance dose of 5 g intramuscularly every 6 h	Blood serum by peripheral vessel; At 24 hours after delivery	1.35±0.42	–
	Term infants, ≥38 weeks (43); Mothers with preeclampsia	Primary 15g injection of $MgSO_4$ (10 g intramuscularly; 5 g intravenously), followed by maintenance dose of 5 g intramuscularly every 6 h	Blood serum by peripheral vessel; At 24 hours after delivery	1.22±0.36	–

* median; † 95% confidence interval. C-section: cesarean delivery; SD: standard deviation. Bold indicates inclusion in the meta-analysis. Magnesium concentrations reported in non-SI units have been converted to mmol/L as follows: mg/dL × 0.411; mEq/L × 0.5
